# Improving Medication Information Presentation Through Interactive Visualization in Mobile Apps: Human Factors Design

**DOI:** 10.2196/15940

**Published:** 2019-11-25

**Authors:** Don Roosan, Yan Li, Anandi Law, Huy Truong, Mazharul Karim, Jay Chok, Moom Roosan

**Affiliations:** 1 Western University of Health Sciences College of Pharmacy Pomona, CA United States; 2 Claremont Graduate University Claremont, CA United States; 3 Keck Graduate Institute School of Pharmacy Claremont, CA United States; 4 Chapman University School of Pharmacy Irvine, CA United States

**Keywords:** visual perception, adverse drug event, human factors design, mobile health

## Abstract

**Background:**

Despite the detailed patient package inserts (PPIs) with prescription drugs that communicate crucial information about safety, there is a critical gap between patient understanding and the knowledge presented. As a result, patients may suffer from adverse events. We propose using human factors design methodologies such as hierarchical task analysis (HTA) and interactive visualization to bridge this gap. We hypothesize that an innovative mobile app employing human factors design with an interactive visualization can deliver PPI information aligned with patients’ information processing heuristics. Such an app may help patients gain an improved overall knowledge of medications.

**Objective:**

The objective of this study was to explore the feasibility of designing an interactive visualization-based mobile app using an HTA approach through a mobile prototype.

**Methods:**

Two pharmacists constructed the HTA for the drug risperidone. Later, the specific requirements of the design were translated using infographics. We transferred the wireframes of the prototype into an interactive user interface. Finally, a usability evaluation of the mobile health app was conducted.

**Results:**

A mobile app prototype using HTA and infographics was successfully created. We reiterated the design based on the specific recommendations from the usability evaluations.

**Conclusions:**

Using HTA methodology, we successfully created a mobile prototype for delivering PPI on the drug risperidone to patients. The hierarchical goals and subgoals were translated into a mobile prototype.

## Introduction

### Background

Adverse drug reactions (ADRs) account for 4.2% to 30% of hospital admissions in the United States, costing up to 30.1 billion dollars annually [1,2]. For decades, the US Food and Drug Administration (FDA) and drug manufacturers have administered detailed patient package inserts (PPIs) with prescription drugs to communicate crucial information for patient safety. In order to comply with FDA regulations, pharmaceutical manufacturers developed PPIs to assist health care professionals and patients in identifying any potential health conditions that may arise when consuming the medications. PPIs provide important information, including warnings, precautions, and lists of adverse reactions and drug interactions [3].

PPIs play a pivotal role for patient safety. The current format for presenting important information in the PPIs does not engage patients in an effective manner. As a result, patients are often unable to identify crucial warnings, the consequences of which are partially manifested in the dramatic increase in ADR-related hospitalizations over the past decade (ie, 117% increase in medication- and drug-related hospitalization from 1997-2008) [4,5]. Moreover, lawsuits and litigation have resulted from off-label use of drugs and drug side effects even when the information to prevent these problems was included in the PPIs.

Recent research suggests minimal patient engagement with PPIs. For example, a recent study explored self-reported drug risk reading by comparing the results of eye tracking and actual information recall. Although the majority of participants claimed to have read the risk information, eye-tracking measures revealed no risk reading, and actual information recall was minimal recall [6]. One plausible explanation, as suggested by the cognition literature, is that patients do not retain medication information due to complex cognitive processes.

The cognition literature offers two perspectives for such behavior: information avoidance and familiarity. Information avoidance refers to not wanting to know information that will cause uncomfortable conflict in the individual’s mind [7]. Familiarity increases with experience frequency and reduces the likelihood that laypeople will look for information. With no negative consequences for repeated usage, individuals become less concerned about the risks associated with the product [8]. Innovative interactive visualizations, such as infographics, have been frequently used to deliver health information due to their ability to present complex data in a simple and clear manner [9,10]. Presenting PPI information using interactive visualization can actively engage patients to reduce information avoidance due to a sense of perceived familiarity and empower patients to retain more information [11].

There is a clear need to present the PPI information in a logical and quick-to-find manner to effectively educate patients about pertinent and crucial life-saving information. Visualizing information using heuristics has proven to help clinicians and patients understand complex scientific data and improve performance decision outcomes [12,13]. Many studies have confirmed that visualization can improve a person’s ability to remember and recall information [14,15]. Heuristics helps reduce cognition overload and cultivates faster ways of processing information by human minds [10]. Thus, it is crucial to organize information in the PPIs in a methodical manner to facilitate heuristic reasoning.

Current estimates suggest more than 40,000 mobile health (mHealth) apps are in use today [16]. Delivering PPI information through the most common platform can reach most patients and maximize the impact of our study. However, mHealth apps proliferate with little evidence for their effectiveness and little support for understanding how to best design these apps [17]. Most mHealth apps present medication information using static texts, and if visualization is used, it is not interactive.

Furthermore, evidence from recent studies shows that many of the mHealth apps do not use human factors design methodologies [18-20]. Without a clear understanding of the end-user requirements, crucial information about health can be presented in such a way that it can be misleading or misread. Among different human factors design methodologies, hierarchical task analysis (HTA) has been widely used for systems design in many different fields and shown to improve overall user satisfaction [21-23]. HTA focuses on the concept of the goal as a unit of behavior in terms of its objectives, which are decomposed into hierarchical subgoals [24]. Although HTA has been rarely used in mHealth app design, many successful fields such as aviation and the military have used HTA for their systems design and decision support.

This research seeks to bridge the gap between drug information presentation and mHealth app development. More specifically, we propose an interactive visualization approach to deliver PPI information to patients in a manner that aligns with their information processing heuristics through a mobile app that uses human factors design methodologies.

### Objective

The objective of the paper is to explore the feasibility of designing an interactive visualization-based mobile app prototype using the HTA approach.

## Methods

### Drug Selection

For the purpose of the prototype, the research team selected a drug product, risperidone, that has applications and uses for children, adults, and the elderly. Risperidone is an atypical antipsychotic drug that is a widely used benzisoxazole derivative approved by the FDA for the treatment of schizophrenia in 1994, for the short-term treatment of the mixed and manic states of bipolar disorder in 2003, and for depression and the treatment of irritability in children with autism in 2006 [25]. Risperidone has several important side effects of which the patients must be aware that are often ignored.

### Hierarchical Task Analysis Construction

HTA is used to understand cognitive task complexity when patients interact with the PPI information and how they would manage these tasks [11,26,27]. To carry out a task to find information, the operator has to go through a logical information scent [28]. Information scent is a term derived from the information foraging theory, which explains human information-seeking and sense interface. Information scent refers to information seekers following hints as a form of either visual or textual context in search of the desired information [23,29,30]. A strong information scent can convince users that they will find what they are looking for at the end of the journey. Information scent is the subjective perception of the cost and value of the sources obtained from proximal cues such as icons (eg, links representing content sources). Thus, placing cues that correspond with the goals can help the information seekers to find relevant information quickly and easily. The output of HTA gives app designers a better understanding of how to place information cues for better information scent.

**Figure 1:**
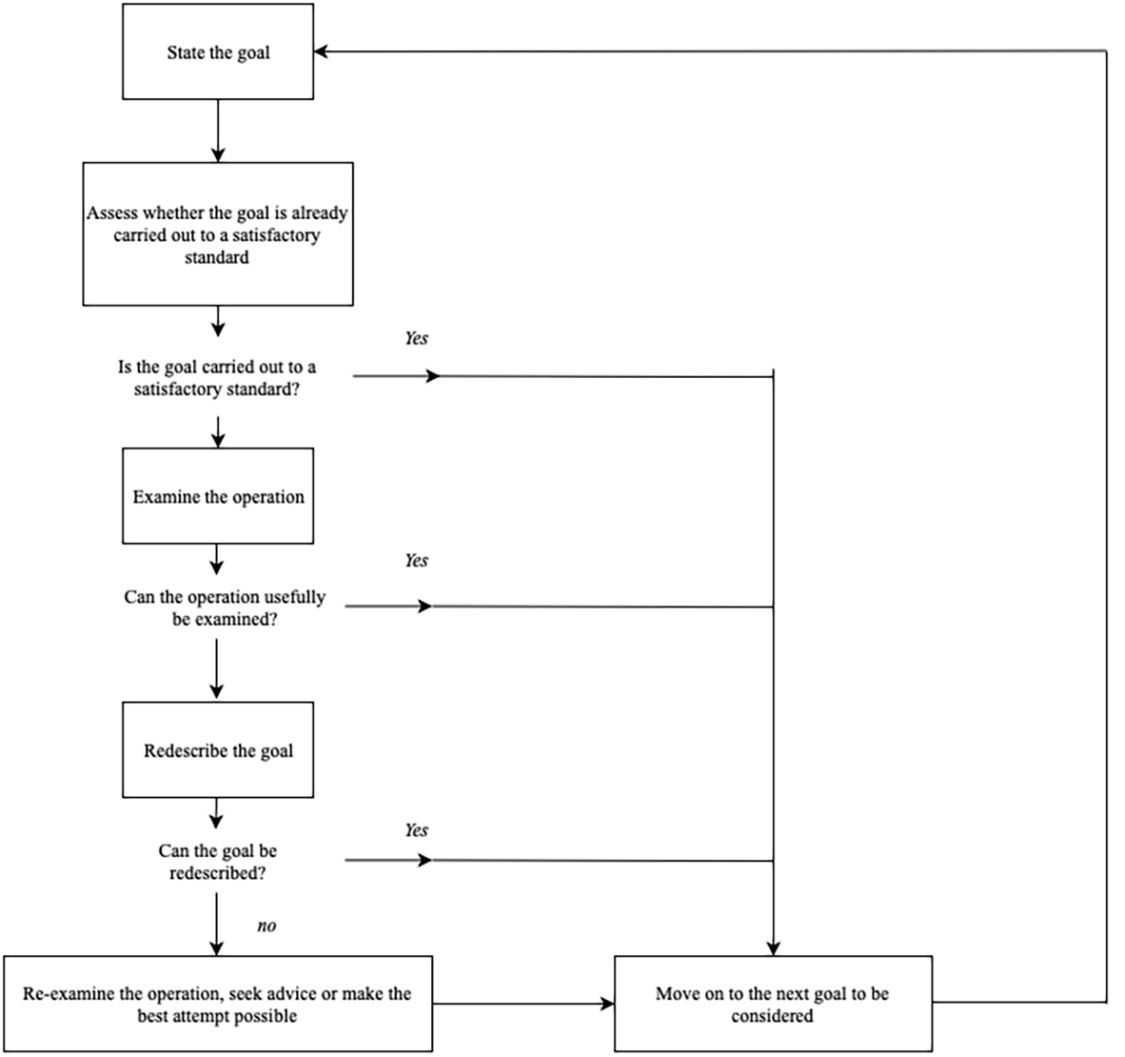
The basic decision-making process cycle for hierarchical task analysis.

We developed 6 high-level information-seeking goals for risperidone based on the 6 types of crucial information two licensed pharmacists decided patients should know about their medications including uses, warnings and precautions, how to take, side effects, how to store, and dosage information contents [31]. Two researchers with backgrounds in pharmacy constructed an HTA map using the goals for each level of crucial information using the HTA methodology. The main strategies for HTA construction are described in Figure 1. After each HTA step was constructed in detail, the two researchers met, discussed, and iterated through the subgoals until consensus was reached. If the two researchers could not agree on any subgoals, the conflict was resolved by the third researcher. The three researchers would then meet and discuss the goals, subgoals, and necessary changes until the action plan for all tasks was clear.

### Interactive Visualization Creation

Once the HTA goals, subgoals, and steps were created, the three researchers met and created the infographics for each step. Each step embeds important medication information related to the 6 goals. The main objective of the infographics is to present critical medication information to patients that is easily understood and does not require a substantial amount of reading. The researchers used a Gestalt approach for the colors, sizes, and fonts of the design [32].

### App Creation

We used Axure RP version 9.0669 (Axure Software Solutions) to prototype and wireframe our ideas into infographics and iterated through the design cycle until the design was approved by the research team [33]. We created infographics for the 6 subgoals and embedded relevant medication information.

Once the prototyping was developed, the functional requirements were coded using Java and PHP: Hypertext Preprocessor. The functionalities were tested several times to ensure proper functioning of the graphics and that links lead to the desired locations. In addition, we assessed our links and hyperlinks to ensure all functionalities were appropriately executed.

### Usability Evaluation of the App

We conducted a usability evaluation of the app with 24 pharmacy students. An exemption was obtained from the Keck Graduate Institute institutional review board and consent forms were signed by the participants. The usability evaluations included two steps. For the first step, we used the concurrent thinking aloud technique to understand and measure the initial reactions of the participants. Each participant was asked first to navigate through the site for 3 to 5 minutes. After initial navigation, participants were asked to think aloud while surfing the interface. All verbal responses were recorded and transcribed. Then two researchers coded recommendations for future iterative design based on the transcripts. These recommendations were further organized under a common theme. For the second step, a System Usability Scale (SUS) survey was administered to 6 participants. SUS is a simple Likert-based scale with 10 statements that examine the global view of the subjective assessment of a user interface. A final SUS score represents a composite measure of the overall usability of the system [34].

## Results

### Hierarchical Task Analysis Construction

**Figure 2:**
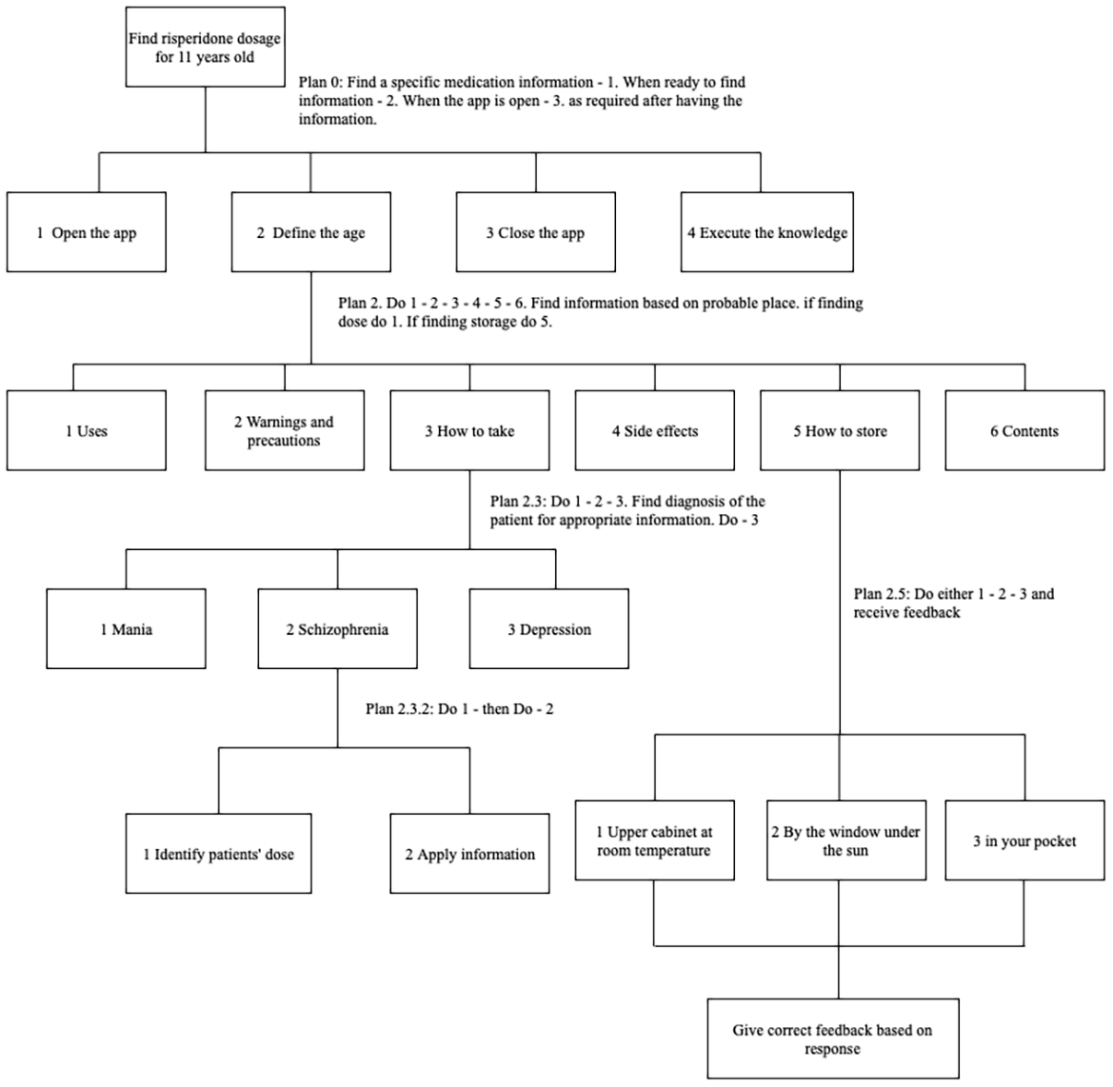
Steps of hierarchical task analysis.

Six extensive steps were developed using the HTA methodology. These steps were later translated into functional requirements in Axure for mockups using an infographics approach. The final prototype was developed after the mockups were verified and reiterated with refinements. We describe two steps that were created using the HTA method in Figure 2. For example, if the high-level goal is to find information on the dosage for an 11-year-old patient, steps would include opening the app, defining the age, and finding the information. After the information requirements are completed, users may close the app and apply the information (eg, giving the medication). After defining the age, subgoals can be found through the app. The information scent requirement leads users to the how to take stage, where 3 indications for the drug (mania, schizophrenia, and depression) are displayed. If the goal is to find the dosage for schizophrenia, the steps would be identifying patient dosage and displaying this information. Similarly, if the goal is to produce information on how to store the medication, plan 2.5 (Figure 2) will show how different interactions can help users find information using a trial-and-error approach. Additionally, it satisfies one of our subgoals of providing feedback in an adaptive fashion (ie, gives user feedback based on their response).

**Figure 3:**
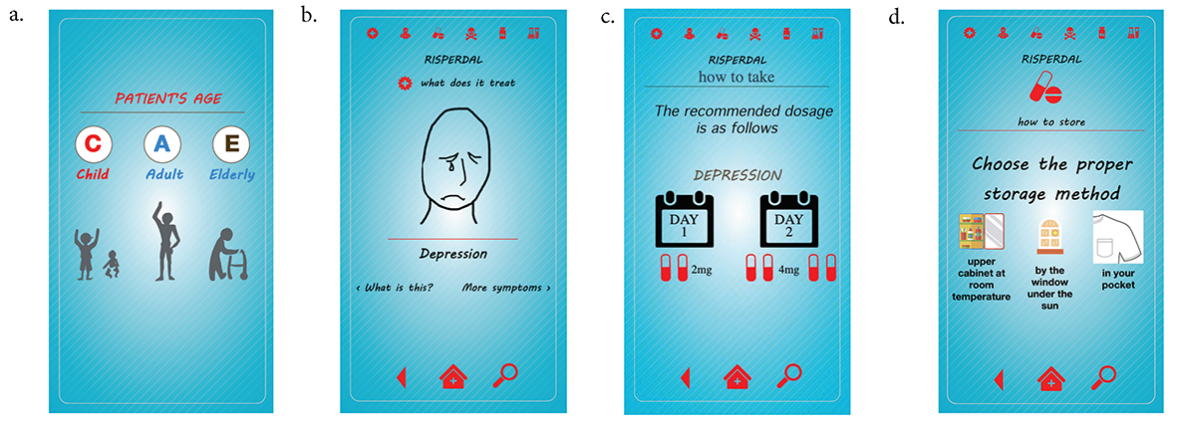
Screenshots from the mobile app: (a) screen showing age-based differences, (b) graphical representation of depression, (c) dosage for depression, (d) screen in which users are asked to choose the proper storage method for the medication.

Explicating steps for goals and subgoals helped the design of step-by-step infographics, as shown in Figure 3, that were instantiated in the mobile prototype. For example, a user who takes Risperdal (risperidone) in Fig 3d is asked to select the proper storage method (in this case, the correct answer is in the upper cabinet at room temperature). If the user selects any other answer, the screen tells the user that the selected answer is wrong and provides an option to choose again until the user chooses the correct answer. This scenario shows how users can interact with our app.

### Usability Evaluations

The specific usability recommendations coded by researchers were categorized into 5 themes: initial impression, problems with page navigation, information presentation, convenience of finding information and significant changes needed. The results are summarized in Textbox 1.

### System Usability Score Analysis Units

The system usability score was computed based on the SUS survey (described in the Methods section) responses. The results (Multimedia Appendix 1) show the composite measure of usability is 74.5% (SD 3%).

**System usability themes and recommendations.**

Initial impression:

- Create a name for the app, Medinfo, and add a description
- Create a logo that brands it as a medication information app
- Create consistent color coding

Problems with page navigation and surfing the site:

- Add search function
- Add file crawling options
- Change pictures to one pill and keep dosing directions

Information presentation, relevance, and positive impression:

- In the how to store section, make the interaction quiz more obvious
- Consider live emoji functions

Convenience of finding information:

- Different font sizes used throughout the app to focus on relevance

Significant changes needed:

- More interactions with the app
- Relevance of warnings and side effects should be clearer

## Discussion

### Principal Findings

Existing designs for drug medication information presentation usually include a snapshot of all related information in simple text format, which often causes information overload [35]. In contrast, a stepwise approach to include safety information has been shown to improve medication information recall for patients [36]. Our innovative approach using HTA to decompose information-seeking tasks and incorporate innovative infographics provides a unique perspective on how to present medication information to patients. More importantly, our research on using HTA corresponds with previous research in which the human factors design approaches proved to be effective [21,37-39].

The three design goals of our mobile app prototype were interaction, information overload reduction, and an enjoyable experience for the patient. Patients should be able to interact with the information. For example, if medicine X should be stored in the refrigerator, patients would be able to click on a picture of the refrigerator under the storage function and get positive feedback based on their response. In this way, patients will feel empowered to seek information. To reduce information overload, only relevant information will be displayed. For example, patients can select only certain aspects of medication information such as how to use to understand different ways to take the medication. The interactive infographics are designed to provide a fun and enjoyable experience for the patient. Instead of using conventional images, we designed the mobile prototype using infographics to visualize the medication information, how to take the medication, and the storage site of the medication. It allowed us to deliver very complex medication information in a fast and understandable way. Additionally, we chose illustrations such as cartoon-type characters and animations, which have been shown to have positive effects on information retention [14].

The study provides novel insights on designing future medication information delivery systems for private and government organizations. Our design can provide insights into digital decision-support design also [40]. It addresses the limitation of the current medication information delivery system design, which does not ensure patient understanding and retention of crucial medication information. According to prior research, the primary causes for missed medications are forgetfulness, discordance between the patient and physician, inability to recall information, and unfavorable side effects [41]. Our research attempts to improve the information recall of patients through the interactive visualization design. We assume that by improving patient medication information recall, the system will improve medication adherence and reduce serious/severe side effects (including adverse events) that burden our health care systems. We thus hypothesize that if patients are more aware of the potential side effects and adverse events, there is an increased likelihood of better adherence to medication management. Future research will test this hypothesis using our prototype system in an experimental setting to evaluate usability features.

Our design has several implications for PPI design. The infographics content we used can be developed for individual medications using the HTA methodology. Companies may be able to have their customers and patients focus more on the life-saving drug label information that otherwise gets ignored. Thus, this process can help with reducing adverse events, monitoring for side effects, and saving industry millions of dollars in future litigations.

Several improvements for our mobile app development have been planned based on the usability recommendations from Textbox 1. One significant change recommended is to make information about warnings and side effects clearer. While currently there is not a standard way to present medication information in a graphic format, one prominent study created an iconic language called Visualization of Concepts in Medicine (VCM) to present medical concepts graphically [42]. The VCM language was designed to present textual information described in drug monographs using only a small number of graphical primitives and combinatory rules. Although the VCM language was initially designed for medical practitioners to remember drug properties, it could be extended to represent warnings and side effects in medication. More specifically, simple sentences about drug warnings, interactions, and side effects could be built with VCM icons.

Second, a reminder function will be added to create more interactions between patients and the app. To be effective, reminders should combine different modalities, including subtle status bar notifications, and should allow users to select alert types that suit their needs depending on their capabilities and social context [43,44]. Design and implementation of the reminder function could continue the same human factors approach we adopted for the current version of the prototype system.

Last, we plan to investigate other ways that would give users more interaction with the app. Designing a game-based app that is generic and aims to change the way patients think and the decisions they make about their health care can be useful [45]. Limited research has examined gamification and its impact on medication adherence. We assume built-in gamification within our mobile app would provide users more time to process the medication information, which would lead to improved medication information recall and better medication adherence.

### Limitations

Our study has several limitations. First, the prototype was designed only for an oral medication. Creating similar designs for other dosage forms such as injectables may be different. Second, the prototype needs to be tested in a real-world setting to verify its effectiveness. In this study, we created a prototype that is not for actual deployment. Our goal in the future is to include verified VCM image icons in the final design during actual implementation. Third, current prototype features such as color, background, and infographics are designed based on the feedback from the research team experts and a limited number of end users. We have demonstrated that it is feasible to design an interactive visualization-based mobile app using the HTA approach. In the next step, we plan to develop the actual mobile app using human factors design with iterations, incremental feedback, and robust testing. Therefore, our final interface design may have different and improved color contrast and background. Fourth, although interacting with simple graphical information may improve critical information recall, we have not tested information recall in patients. However, we plan to investigate other ways to increase patient interaction with the medication information and test recall in future.

### Future Work

Once our mobile app is improved and deployed, we plan to conduct an experiment that compares medication information recall between patients who interact with PPI information through our mobile app versus patients who receive PPI in a paper format. The experiment would allow us to demonstrate the effectiveness of delivering PPI information via interactive infographics on a mobile app.

### Conclusions

In this study, our goal was to design an interactive infographics-based medication information delivery system to reduce information overload and improve medication information recall. Using the HTA methodology, we successfully created a mobile prototype for delivering PPI for the drug risperidone. The hierarchical goals and subgoals were translated into a mobile prototype.

## Appendix

Multimedia Appendix 1 System Usability Scale scores.

## Abbreviations

ADR: adverse drug reaction
FDA: US Food and Drug Administration
HTA: hierarchical task analysis
mHealth: mobile health
PPI: patient package insert
SUS: System Usability Scale
VCM: Visualization of Concepts in Medicine
