# Supplementary figures and images for: Improving Medication Information Presentation Through Interactive Visualization in Mobile Apps: Human Factors Design
Source: JMIR Mhealth Uhealth. 2019 Nov 25;7(11):e15940. doi: 10.2196/15940 (PMC6902132; doi:10.2196/15940)

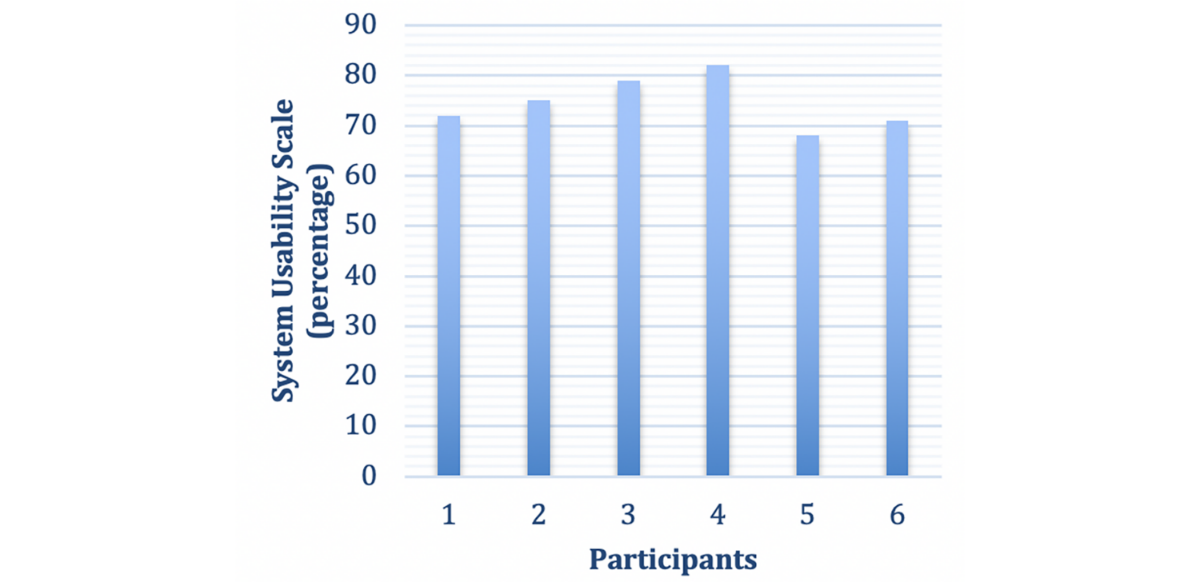

Supplement: Multimedia Appendix 1 [file mhealth_v7i11e15940_app1.png]
